# Pathway level metabolomics analysis identifies carbon metabolism as a key factor of incident hypertension in the Estonian Biobank

**DOI:** 10.1038/s41598-025-92840-w

**Published:** 2025-03-12

**Authors:** Liis Hiie, Anastassia Kolde, Natalia Pervjakova, Anu Reigo, Estonian Biobank Research Team, Mait Metspalu, Andres Metspalu, Lili Milani, Tõnu Esko, Erik Abner, Urmo Võsa, Tõnu Esko, Krista Fischer, Priit Palta, Jaanika Kronberg

**Affiliations:** 1 Estonian Genome Centre, Institute of Genomics, University of Tartu, Riia 23b, 51010 Tartu, Estonia; 2 Institute of Mathematics and Statistics, University of Tartu, Narva 18, 51009 Tartu, Estonia

**Keywords:** Metabolomics, Biobank, Incident disease, Data-driven, Pathways, Hypertension, Metabolomics, Hypertension

## Abstract

The purpose of this study was to find metabolic changes associated with incident hypertension in the volunteer-based Estonian Biobank. We used a subcohort of the Estonian Biobank where metabolite levels had been measured by mass-spectrometry (LC-MS, Metabolon platform). We divided annotated metabolites of 989 individuals into KEGG pathways, followed by principal component analysis of metabolites in each pathway, resulting in a dataset of 91 pathway components. Next, we defined incident hypertension cases and controls based on electronic health records, resulting in a dataset of 101 incident hypertension cases and 450 controls. We used Cox proportional hazards models and replicated the results in a separate cohort of the Estonian Biobank, assayed with LC-MS dataset of the Broad platform and including 582 individuals. Our results show that body mass index and a component of the carbon metabolism KEGG pathway are associated with incident hypertension in both discovery and replication cohorts. We demonstrate that a high-dimensional dataset can be meaningfully reduced into informative pathway components that can subsequently be analysed in an interpretable way, and replicated in a metabolomics dataset from a different platform.

## Introduction

Hypertension is a chronic medical condition affecting more than one billion people worldwide^1^. Its prevalence has been estimated to be 32% in women and 34% in men among adults between ages 30–79 in 2019^2^. It is also the most important preventable risk factor for cardiovascular disease, mortality and all-cause morbidity^3^. Hypertension risk can be reduced by healthy lifestyle^4,5^ and also treatments are available. To understand disease development, it would be crucial to identify the earliest molecular changes preceding the diagnosis. Once the disease has onset, the molecular state of the organism, characterised by various omics levels, may already reflect the concomitant effect of the disease or treatments. Capturing the earliest changes before diagnosis might also shed light on possible mechanisms and provide directions for further research for prevention.

Previous hypertension research has focused on both traditional variables and, in recent years, also on different omics data layers. For example, body mass index^6–8^ and dietary amino acids^9,10^ have been shown to be associated with predisposition to hypertension. Omics data modalities make it possible to study hypertension in a data-driven manner, considering thousands of variables. For example, GWAS studies have found SNVs associated with hypertension^11,12^ and have led to the development of polygenic risk scores tailored to hypertension prediction^13^. Differential gene expression patterns have been studied in both animal models^14^ and humans^15^. Additionally, urinary proteomics has provided insights into proteomic differences between salt-sensitive and salt-resistant newly hypertensive patients^16^. Metabolomics, including both mass-spectrometry and ^1^H-NMR based metabolite profiling has also helped uncovering understanding the development of hypertension^17–19^. Multiple data sources, such as GWAS results, protein-protein interactions and annotated pathway databases have been used for network-based analysis^20^. Mendelian randomisation studies have also shed light into the potentially causal proteins^21^ or metabolites^22^.

Biobanks, with regular follow-up from electronic health records (EHR) and with some participants developing diseases after joining, are an ideal resource for studying the earliest changes before symptoms or diagnoses. For example, blood pressure progression was studied in 504 people in Sweden with 5-year follow-up and validated in another cohort of 222 people^23^, using the Metabolomics platform and constituting the first study to use longitudinal blood pressure progression using global metabolomics. In a later Swedish study of 2278 individuals, Metabolon data was used for the prediction of incident cardiovascular disease^24^ in a population-based sample, where 107 individuals were diagnosed a cardiovascular disease during the follow-up period of almost 10 years. The results were validated in a separate cohort in Sweden. These two studies, both from Sweden, show the power of good quality EHR for understanding the early changes leading to a disease. In another study in the TwinsUK cohort, incident hypertension (989 cases and 1628 controls) was studied in respect to glycosylation profiles and found 6 glycan traits associated with incident hypertension, with replication in 10001 Dalmatians (106 individuals) and KORA (729 individuals)^25^. Proteomics has also been used to study incident heart failure. For example, plasma proteome of over 4800 proteins was measured in 13900 individuals in ARIC and HUNT cohorts in the US and Norway, from whom 1570 had a heart failure event during the follow-up time. Proteomic analysis identified 39 proteins associated with incident heart failure^26^. The difference, besides the utilisation of different omics layers, is that the ARIC cohort, recruited between 1987 and 1989, relies on examinations for health status, with participants attending up to 5 visits until 2013. The HUNT study participants provided initial self-reported diagnoses at recruitment in the 1980s, however their current health status is updated via linkage to EHR, making it a valuable resource with a follow-up of almost 40 years^27^.

Although previous research has identified metabolites and proteins associated with hypertension, several challenges remain. The first challenge is the number of samples, which is usually much lower than the number of variables. It has been suggested that a “one in ten” rule should be used for predictive modelling, meaning that one variable could be used for every 10 events^28^. In survival models, this would be the number of uncensored events. However, this 1 in 10 rule is not generally followed, for example, from 118 studies evaluated, only 4 followed the 1 in 10 rule and one followed 1 in 5^29^. This poses a challenge for the use of omics studies as these usually contain many features. For example, for the Metabolon platform, the required sample size for 1500 assayed metabolites would be 15000 individuals. The second challenge lies in interpreting the results effectively. Despite identifying metabolites associated with the studied disease, the interpretation is frequently complicated. For instance, in^24^, although several metabolites are linked to hypertension, the use of tools like MetaboAnalyst^30^ assigns each metabolite to just one pathway, making it difficult to determine which pathways might be implicated. Thirdly, in different studies and platforms, the metabolites identified can be different and non-overlapping, making comparison of single metabolites or replicating studies between different platforms challenging.

The aim of the study was to identify metabolic pathways associated with incident hypertension while addressing all of the challenges described. We utilized a Metabolon metabolomics dataset comprising 1,505 metabolites measured across 989 individuals to identify metabolites associated with the future development of hypertension, defined from the EHRs of universal healthcare in Estonia and covering both diagnoses and prescriptions. Of 989 people, 101 developed hypertension after the sample was taken. We overcome the challenge of a large number of metabolites compared to the sample size by reducing the dataset into pathway-based principal components, which are then used in survival models instead of single metabolites. Pathway-based approaches have been used before for gene expression^31–34^ and multi-omics integration^35^. The major advantage of pathway-based dimensionality reduction has been suggested to be improved statistical power and enhanced biological interpretability^32,34,35^. Meaningful and biologically interpretable pathway components also solve the challenges single metabolites would have for pathway-based enrichment analysis. Pathway-based approach also simplifies cross-platform comparisons at the pathway level. We identify increased body mass index (BMI) and changes in the carbon metabolism pathway as risk factors for future hypertension. We show that pathway-based reduction of a large set of metabolites provides biologically interpretable results which are consistent between two different datasets of different platforms in the Estonian Biobank. The current study discovered potential early changes leading to hypertension and highlights the power of available omics data from biobanks and regular follow-up via electronic health records.

## Results

### Pathway-based approach to study incident hypertension in a volunteer-based biobank

**Figure 1:**
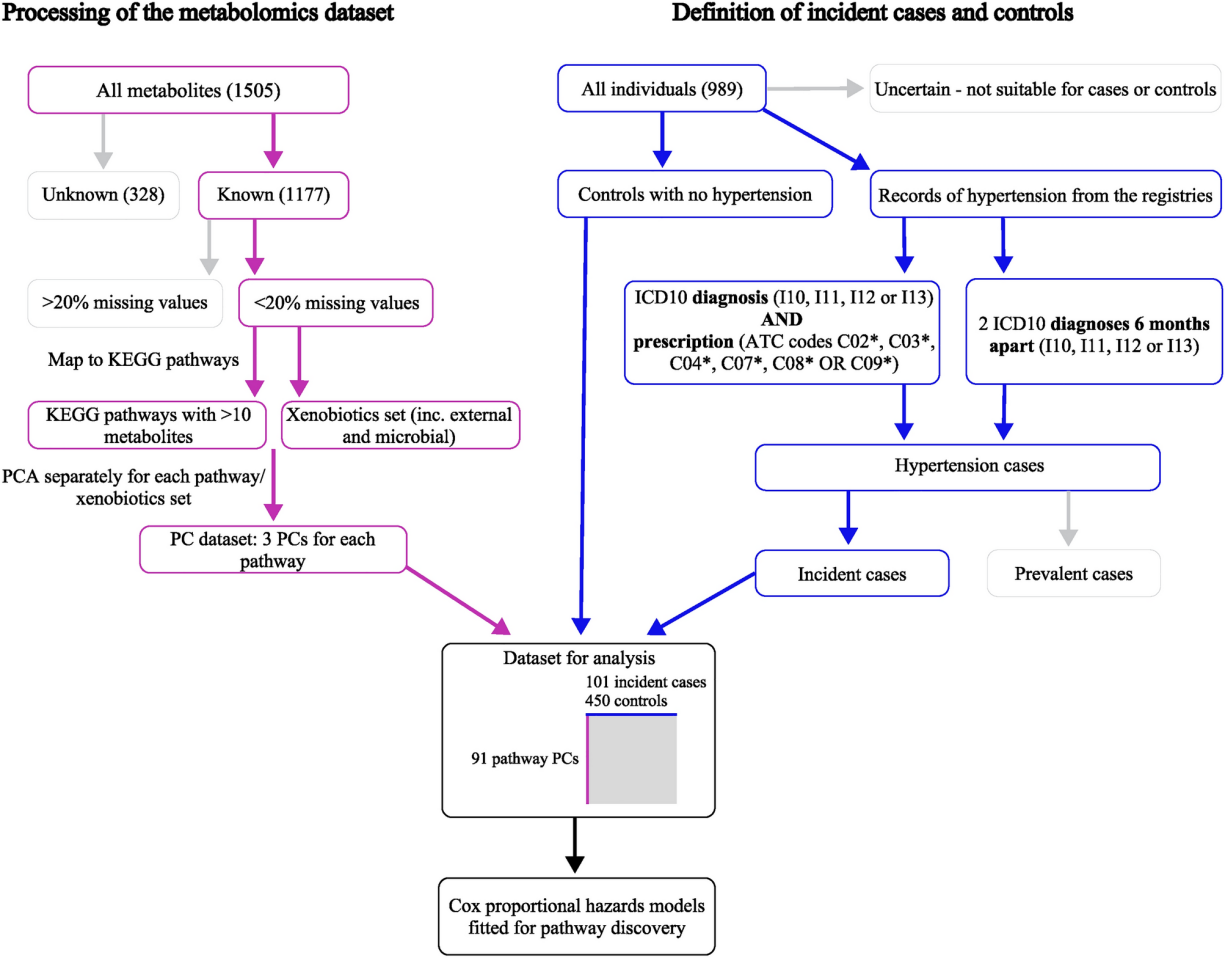
Approach overview. Metabolon dataset of 1505 metabolites was reduced to 91 KEGG pathway components to be used for survival modelling of incident cases and controls defined based on electronic health records in the Estonian Biobank.

Hypertension is a highly prevalent disease, and thus, we explored whether pathway-based analysis of a population-based sub-cohort of 989 individuals could give insights into the early changes before hypertension. We developed an approach to overcome two limitations in the analysis of metabolomics datasets: the pathway-based interpretation of results and the problem of large number of features compared to the sample size. Our approach overview is shown in Fig. 1. Briefly, we used KEGG pathways^36–38^ to reduce the dimensionality of the dataset from 1505 metabolites to 91 pathway principal components. We defined the incident cases and controls based on ICD-10 diagnoses and ATC-based prescriptions from electronic health records that are regularly linked to Estonian Biobank participants. The newly prepared dataset was then used in survival models with additional covariates.

### Mapping metabolites into KEGG pathways to aid dimensionality reduction

First, we used KEGG (Kyoto Encyclopedia of Genes and Genomes,^36–38^) IDs of all metabolites of the dataset (1055) to map them into KEGG pathways. Our first question was to decide which pathways to include based on the number of metabolites in the pathway. The number of pathways and metabolites in the pathway and metabolites from the KEGG dataset in the pathway is shown in Table S1. We excluded the superpathway “Metabolic pathway” (247 KEGG codes) as it included smaller pathways present in the dataset. In addition to this, the analysis excluded nucleotide metabolism (hsa01232) since its two main sub-pathways, purine metabolism (hsa00230) and pyrimidine metabolism (hsa00240), share the same metabolites and were already included.

Nine KEGG pathways included $>20$ metabolites, 17 consisted of at least 15 metabolites, and 33 were at least 10 metabolites. We choose to include pathways with at least 10 metabolites in the analysis. These 33 pathways have 255 unique metabolites identified with KEGG codes. Of these 255 metabolites, 213 are present in the metabolomics dataset that belong to 33 different pathways. The following analysis will use these 33 pathways.

Metabolites annotated as xenobiotics (except one metabolite, sulfate (CHEMID 100002528, KEGG C00059)) were not assigned to KEGG pathways. The xenobiotics in the Metabolon dataset include microbial metabolites, metabolites from food components, tobacco, and chemicals from the environment, such as perfluoroalkyl and polyfluoroalkyl substances (PFAS). As the Metabolon dataset also contains xenobiotic metabolites from microbial metabolism, food, and environmental sources, in addition to endogenous metabolites from human pathways, we decided to treat all chemical xenobiotics (microbial xenobiotics were not included) as an additional group in the following analysis steps, combining them into a “xenobiotic set” (as most xenobiotics did not map into the human pathways considered for analysis) to be used in the PCA.

### Creation of a pathway-PC dataset

Secondly, we performed principal component analysis on 33 pathways and the xenobiotic set. We considered three first PCs, checking the cumulative % variance explained. The results are shown in Table S2. If the cumulative % variance explained is $<5\%$ comparing 1st and 2nd or 2nd and 3rd component, we excluded the latter component from further analysis. Only 1 PC was removed in this way. Only one pathway (Biosynthesis of unsaturated fatty acids) would be represented by 2 PCs, and all others would have 3 PCs. Interestingly, the cumulative variance explained shows that more than three PCs could represent the xenobiotics class. It is a diverse set of metabolites from different sources. We decided to use only 3 principal components also for this set to follow the rules defined for the KEGG pathways, some of which also would need more than 3 PCs to explain most of the variance. As the number of samples is small compared to the number of variables, keeping the number of variables close to 10% of the sample size would be desirable. Moreover, because we intended to make all decisions based on data, we would also need to increase the PCs for other pathways, increasing the number of variables.

Principal components of one pathway cannot be correlated, but PCs of different pathways can. To remove highly correlated pathway components, we calculated correlations between all components and in case of correlation $>0.9$, the component from the smaller pathway was excluded from the analysis. The highly correlated pathways are shown in Table S3.

### Incident hypertension can be studied using volunteer-based samples

We defined hypertension cases based on ICD-10^39^ codes from electronic health records from primary care and hospitals and ATC codes from electronic prescription data. Briefly, cases were defined as either two diagnoses of hypertension at least six months apart or a diagnosis and a prescription for hypertension. Detailed case definitions are shown in Table 4. Individuals who had only one diagnosis of hypertension from the EHR or an elevated blood pressure reading but no diagnosis were classed as uncertain and excluded from cases and controls. Detailed case definitions and exclusion criteria are described in the methods section. Cohort characteristics of the subset of the Estonian Biobank participants who had Metabolon data available is shown in Table 1. By analysing the electronic health records of 885 people who had covariate data available (from the initial 989), 334 had hypertension diagnosis before joining the biobank (prevalent cases), 22 had some diagnosis but not sufficient evidence (uncertain), 101 would develop hypertension during the follow-up (incident cases), based on inclusion criteria of hypertension, and 450 had never had a hypertension diagnosis before joining and would not have until the end of follow-up (controls).

**Table 1:** The descriptive statistics of Metabolon subcohort of the Estonian Biobank. The statistical significance of differences between incident cases and the control group is assessed using the Welch Two Sample t-test for numeric variables and the Pearson Chi-squared test for categorical variables. A *p*-value less than 0.05 indicates a statistically significant difference between the two groups.

Variable	Prevalent cases	Incident cases (i)	Controls (c)	*p*-value (i,c)
N	334	101	450	
Sex, male (%) Sex, female (%)	44.9 55.1	34.7 65.3	31.3 68.7	0.52
Age at sample (mean±sd)	61±13	48±13	37±13	1.27 $\cdot 10^{-12}$
Average follow-up years		5±4	9±5	3.43 $\cdot 10^{-12}$
BMI (mean±sd)	29.9±5.7	28.2±5.2	24.3±4.7	1.15 $\cdot 10^{-10}$
Smoking, current (%) Smoking, former (%) Smoking, never (%)	14.1 29.6 56.3	28.7 14.9 56.4	23.6 21.3 55.1	0.27
Education, low (%) Education, intermediate (%) Education, high (%)	14.1 56.8 29.1	6.9 56.4 36.6	4.7 45.3 50.0	0.048
Residency category, rural area (%) Residency category, town (%) Residency category, city (%) Residency category, unknown (%)	32.9 9.3 43.4 14.4	39.6 8.9 44.6 6.9	32.0 12.0 49.6 6.4	0.46
Time of day, before 10 (%) Time of day, 10–15 (%) Time of day, after 15 (%)	22.5 54.5 23.1	26.7 49.5 23.8	25.6 54.4 20.0	0.61

### BMI and carbon metabolism are associated with incident hypertension

The final dataset consisted of 91 principal components from 33 different pathways and the separate xenobiotics set, covering 235 metabolites; sample data including age at sample and time of day; hypertension diagnosis data including incidence and prevalence of the cases, age at diagnosis; death data including age at death; censoring data including age at the end of follow-up period; questionnaire data including sex, BMI, smoking status, education level, and residency category. Cox proportional hazards (CPH) models were used to find the pathway components associated with the risk of hypertension, where age was used as the time scale. This approach corresponds to adjusting for age^40^.

#### Baseline model

First, a CPH model was fitted with sex, BMI, smoking status, education class, residency category and time of day included as predictors. The reference levels for categorical variables are “never” for smoking, “male” for sex, “rural area” for the residency category, “low” for the education level and “before 10” for the time of day. The resulting model is shown in Table 2.

In Model 1, where we included sex, BMI, smoking status, education class, and time of day as predictors, the only variable of statistical importance is BMI with a *p*-value of $7.03\cdot 10^{-10}$. All possible models that can be constructed by dropping a single model term were compared, and the Chi-Square test was used to find the variable which, when left out, will leave the best model with the lowest Akaike’s information criterion (AIC). Model 2 remains with only one predictor, BMI. The hazard ratio of 1.10 indicates that the higher value of BMI is associated with an increased risk of hypertension. The model improved, with AIC dropping from 820.66 to 808.71. The proportionality check did not indicate any violations towards CPH model assumptions in either model. Model 2 would be used as a baseline model for pathway discovery.

**Table 2:** Hazard ratio estimations and *p*-values for the fitted Cox proportional hazards baseline models.

	Model 1		Model 2	
	Exp(coef)	*p*-value	Exp(coef)	*p*-value
Sex: female	0.95	0.83		
BMI	1.11	7.03$\cdot 10^{-10}$	1.10	1.34$\cdot 10^{-9}$
Smoking: current	1.30	0.29		
Smoking: former	0.86	0.61		
Education class: intermediate	0.73	0.47		
Education class: high	0.54	0.17		
Residency category: city	1.16	0.52		
Residency category: town	0.87	0.71		
Residency category: unknown	0.63	0.31		
Time of day: after 15	0.77	0.38		
Time of day: between 10 and 15	0.73	0.21		
AIC	820.66		808.71	

#### Pathway discovery

**Figure 2:**
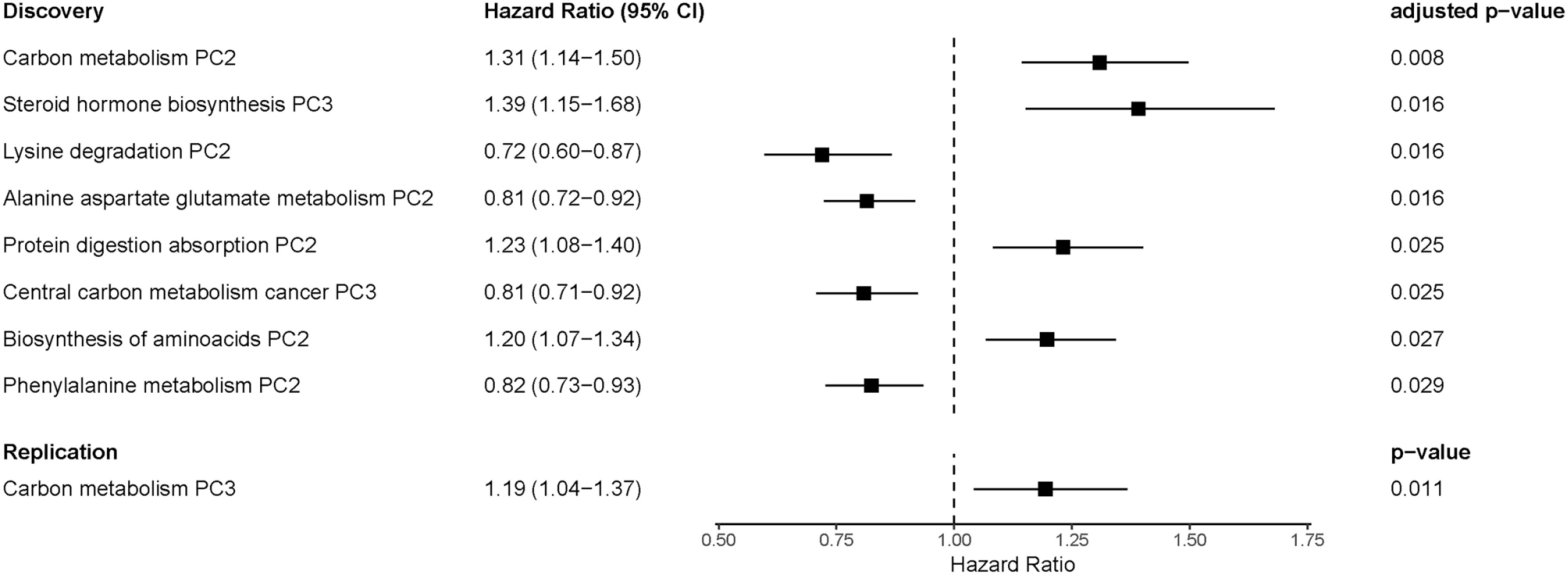
Forest plot of single pathway component models: BMI and each of the 91 pathway components. Significant pathway components are shown for the discovery cohort. For the replication cohort, from 3 carbon metabolism PCs, PC3 was significant and is shown.

A multivariable model was found in the forward stepwise selection manner for the pathway discovery, in a similar manner as has been used before for NMR-based metabolites^40^. First, 91 models were created, including BMI and one of the 91 pathway components as predictors. Results from the models with single pathway components are shown in Fig. 2 and Table S4. The pathway component leading to the smallest Benjamini-Hochberg corrected *p*-value in the CPH model was chosen and included in the prediction model. The process was repeated until no additional pathway components were significant at the Benjamini-Hochberg corrected significance level with FDR controlled at level 0.05.

The first pathway component added to the model was PC2 of carbon metabolism (hsa01200). After that, no other pathway components proved significant. Therefore, the final model consists of BMI and the PC2 of the carbon metabolism pathway, as seen in Table 3. The BMI remains important in the model. The model improved compared to the baseline model and has an AIC of 795.84. The proportionality check did not indicate any violations towards CPH model assumptions in either model.

**Table 3:** Hazard ratio estimations and *p*-values for the final Cox proportional hazards model in the discovery dataset.

	Model 3	
	Exp(coef)	*p*-value
BMI	1.09	1.84 $\cdot 10^{-7}$
Carbon metabolism (hsa01200) PC2	1.31	8.92 $\cdot 10^{-5}$
AIC	795.84	

Therefore, a component of the carbon metabolism pathway can help predict hypertension even years before the onset of the disease. A hazard ratio of 1.31 shows that the higher value of carbon metabolism component is associated with an increased risk of hypertension. We further explored the metabolites included in this pathway and their importance in the component. There are 14 metabolites in the carbon metabolism pathway. These are listed in Table S5 with the respective biochemical class and loading of the metabolite in the second principal component.

### Single metabolite analysis

To compare our approach with single metabolite approaches, we analysed a subset of 212 metabolites that were previously mapped to KEGG pathways. These metabolites were modelled exactly as pathway components, with BMI in the baseline model. After FDR adjustment, there were no significant metabolites. Results are shown in Table S9. Additionally, a full dataset of annotated and unannotated metabolites that are present in $>80\%$ individuals (1055 metabolites) were analysed by creating 1055 separate models with BMI in the base model. After FDR adjustment, there was one significant metabolite, X-21733, which is an unannotated metabolite. However, the model including this metabolite violates the CPH model assumptions, as the proportionality check indicated that the hazards are not proportional. Results are shown in Table S10.

### Replication in a different cohort

We replicated the results of the pathway-PC analysis in an already published dataset^41^ where Estonian Biobank participants, aged 70–79, with no pre-existing hypertension, cancer, CAD, COPD, diabetes, stroke or Alzheimer’s disease, were selected from the Estonian Biobank for LC-MS profiling in the Broad Institute. During the follow-up time of 8–14 years, 187 of the participants died. We extended the follow-up to finish 2021-12-31 based on electronic health records. The characteristics of the replication cohort are shown in Table S6.

In this dataset, there were 74 controls and 154 incident cases of hypertension based on our case definition criteria described earlier for the discovery cohort. We used all non-metabolomic variables as before to create the base model. The pathway model was tested with only the 3 PCs of metabolites overlapping with the carbon metabolism pathway between the two datasets. We ran the analysis as before, creating a baseline model and then running individual models with each pathway component. Because the replication dataset utilized a different metabolome profiling platform than the discovery dataset, we recalculated the PCs in the replication dataset. BMI remained the best predictor in the baseline model ($p=0.0002$), and carbon metabolism pathway PC3 was significant ($p=0.01$). Results are shown in Table S7 and Fig. 2. A hazard ratio of 1.19 shows that the higher value of carbon metabolism component is associated with an increased risk of hypertension. The direction of the association is the same as in the original dataset. The loadings of carbon metabolism PC3 are shown in Table S8. The five metabolites with the highest loadings were aspartate, glutamate, serine, alanine, and citrate/isocitrate. Notably, the metabolites showing the highest loadings in the replication dataset (glutamate, serine, alanine, and citrate/isocitrate) are also among the top 5 loadings on the analysis in the discovery dataset.

## Discussion

Hypertension is a highly prevalent disease that is a risk factor for other diseases, and therefore, it is important to understand the earliest molecular changes preceding the development of the disease. While large biobanks and omics datasets have the potential to discover such early changes, there are technical challenges related to sample size, different platforms, and the interpretation and reproducibility of results.

We have used pathway-based data-driven analysis to study metabolic changes associated with incident hypertension in a volunteer-based biobank. Our approach addresses the challenge of using high-dimensional datasets with limited number of samples. It also overcomes the difficulties of interpretation of results in a pathway context by reducing a large number of variables into pathway principal components, a smaller set of features that still represent aspects of the main pathways. Results based on principal components of KEGG pathways are interpretable and could be replicated in a dataset from a different platform.

We find BMI and metabolites from the carbon metabolism pathway associated with incident hypertension. The BMI has been associated with the risk of hypertension before^5–8,42^, as with many other diseases. Single metabolites from the carbon metabolism pathway have also been associated with hypertension and incident hypertension in both human and animal models. For example, dietary glycine and alanine estimated based on food frequency questionnaires were found to be associated with blood pressure^43^. Relationships between glycine levels and hypertension have also been shown in mice^44^, whilst association with both serine and glycine has been shown in rats^45^. Many metabolites from the carbon metabolism are part of the TCA cycle. TCA cycle has been studied in Dahl salt-sensitive rats with proteomics and metabolomics, showing that high salt diet affected the TCA and also the glycolysis pathway, leading to salt-sensitive hypertension^46^. In humans, TCA cycle (citric acid cyle) has been shown to differ between individuals resistant to spironolactone treatment and responders^47^. In a review and meta-analysis of studies of metabolomics and essential hypertension in both humans and animals, 210 identified metabolites from animal studies were analysed with MetaboAnalyst^48^, resulting in three pathways with high impact, two of which contain metabolites from the carbon metabolism pathway (alanine, aspartate and glutamate metabolism and glycine, serine and threonine metabolism)^49^. Alanine, aspartate, and glutamate metabolism was also identified in analysing 393 human metabolites associated with essential hypertension^49^. In a review of metabolites and hypertension^50^, metabolites in the TCA cycle, namely malate, fumarate, citrate, pyruvate, alpha-ketoglutarate, are all central in the pathway representation of metabolic pathways conversing in hypertension regulation. In the largest study using mass-spectrometry for cardiovascular diseases to date^24^, with a sample size of 2278 individuals, 107 individuals had an incident cardiovascular disease. For these individuals, blood pressure at the start of follow-up was 134.1 and more severe cardiovascular outcomes were studied. However, amino acids alanine, aspartate, glycine, and serine metabolism pathways were identified. For the interpretation of results, MetaboAnalyst^30,48^ was used, but no significant enrichment was found, as each metabolite mapped to separate pathway. In a review of hypertension, many of the metabolites of the carbon metabolism are described from previous studies^51^. These include glycine, alanine, glutamate metabolism, aspartate and pyruvate. However, these results are all shown comparing people with prevalent hypertension and controls. As we find that these metabolites, as part of the carbon metabolism pathway are associated with future hypertension, these metabolites might indicate early changes and could be explored further in future studies, including for example analysing the association of these metabolites with incident hypertension using overlapping metabolites from an NMR-based metabolomics platform. As our results excluded people who had a systolic blood pressure over 140 mmHg or diastolic blood pressure over 90mmHg at the time of joining the biobank and giving the blood sample, making the future hypertension cases likely to be healthy at the time of joining, not people who were in fact already hypertensive but not yet diagnosed. It is especially interesting, that from two pathways highlighted, one of them, alanine, aspartate and glutamate metabolism, in our analysis the PC2 of the pathway was significant in the analysis where single KEGG-components were added individually and PC1 of the same pathway was correlated with carbon metabolism PC1, therefore removed from the analysis. The advantages of pathway-based analysis have also been highlighted in a recent publication of multi-omics integration tool, where pathway-level datasets are shown to have improved predictive performance compared to single-molecule analysis^35^.

The main limitation of our work is the sample size compared to the number of metabolites. However, our approach aims to overcome this and we show that even with a starting sample size of 989 individuals, results are meaningful and replicated in a dataset from a different platform in a study population with different inclusion criteria. The use of a volunteer-based sample could be viewed as a limitation, as there might be people among the controls who for example, have not yet been diagnosed. However, this can also be viewed as an advantage, as biobanks allow the study of incident diseases and with a universal healthcare system and the linkage of both hospital and primary care diagnoses with the biobank, common diseases, such as hypertension are likely to be diagnosed if a person attends for example occupational health appointments or visits the GP. We acknowledge that the true physiological onset of hypertension may precede the date captured by medical records-this is an inherent challenge for most chronic conditions that do not require immediate hospitalization. Nonetheless, using EHR data to identify the earliest recorded date of a hypertension-related event is a widely accepted and practical approach in large-scale studies. This method provides the best available proxy for incident onset in a real-world setting.

Another limitation is the mapping of metabolites to KEGG pathways. As KEGG pathways are based on previous human knowledge and likely contain knowledge gaps, other pathway datasets could have been additionally used. Alternatively, a completely PCA-based or clustering-based approach could have been used, for example clusters could be built from all metabolites, including unannotated ones, using mutual information^52^ or correlation based^53,54^ approaches, enabling the inclusion of all metabolites, even the ones that are not annotated or mapped to pathways. Principal component analysis could then be performed on each cluster and principal components of clusters used instead of KEGG PCs. These clusters would then need to be annotated by enrichment analysis for pathways, but the interpretation of these clusters from the Metabolon dataset would have to also rely on previous knowledge and could make replication on a different dataset and platform more challenging. However, the clusters-based approach would be beneficial for datasets where a large number of metabolites cannot be mapped to pathways, for example for xenobiotics, despite difficulties of cluster annotation. To include metabolites not in KEGG pathways, we include unmapped chemical xenobiotics as a separate group for which principal components are calculated. Ideally, 3 approaches, single metabolites, pathway-based and cluster-based could be used for the same dataset, each with their own strengths and weaknesses: single metabolite analysis as a more traditional way that might not result in any significant metabolites; pathway-based approach as the most suitable for biological interpretation but excluding unannotated metabolites; and cluster-based approach for its strength to include all metabolites, but more difficult to interpret. The main advantage of performing assignment into KEGG pathways before analysis is that in addition to straightforward biological interpretation, it allows the results to be replicated with different metabolomics platforms or even on different omics layers, as long as they can be mapped to KEGG.

The number of principal components could have ideally been chosen based on explained variance of the PCs in the pathway, resulting in more PCs for each pathway. This could be used as a criteria in future studies with much larger datasets, such as NMR-based metabolomics which have been measured in hundreds of thousands of individuals in major biobanks^55^.

As both metabolomics datasets are sub-cohorts of the Estonian Biobank, there can be potential shifts from population-level related to the selection process of these cohorts. However, in the case of the Metabolon dataset, participants were not selected based on diagnoses but a large number of loss of function mutations (Yu et al, in preparation) and in the replication cohort, the participants were selected based on their mortality in the next 10 years after the sample^41^, while age was also used as a selection criterium. Hypertension is a risk factor for other diseases, including cardiovascular diseases which were part of the causes of death, therefore, participants in the two cohorts differ in their prevalent and incident diagnoses and age at the time of the sample. However, despite these differences, the carbon metabolism pathway was identified to be associated with future hypertension in both cohorts.

We show the potential for pathway-based analysis of mass-spectrometry data and EHR-based diagnoses in a volunteer-based biobank to study early metabolic changes preceding the diagnosis of hypertension. Our results are in concordance with previous studies of hypertension in both humans and animals, being the first study using a mass-spectrometry dataset reduced into pathway components to study incident hypertension in a volunteeer-based biobank.

## Methods

### Estonian Biobank

The Estonian Biobank is a population-based biobank with 210,000 participants recruited since 2002^56,57^. Participants in the biobank have signed a broad consent and provided a plasma sample upon enrollment. Additionally, they completed a questionnaire during the enrollment process. Health data are consistently linked from national databases, including the Health Insurance Fund, Cancer Registry, and Death Registry. Health information consists of both ICD-10 codes for diagnoses from primary care and hospitals and prescription data for ATC codes. In the current analysis, diagnoses until 2021-12-31 were used.

### Metabolon data generation

The study population included of 992 participants the Estonian Biobank for whom metabolomics profiles were generated using the Metabolon platform^58^. After the sample collection (2002-2019), samples had been stored at -80 and were sent to Metabolon for metabolite profiling. The Metabolon dataset has been previously described in the contect of comorbidities^59^.

### Data pre-processing

Metabolon provided peak area data with missing values as ’NA’ and volume normalised imputed data. In order to filter out metabolites that are missing in $>20\%$ individuals and to perform our own imputation, fields with NA in the raw data matrix were set to NA in the batch- and volume- normalised matrix (i.e. to replace the imputed values with NA), resulting in a dataset that is volume-normalised but not imputed. Metabolites that had more than 20% missing values were removed from the analysis, leaving a dataset of 1055 metabolites. We intended to remove individuals with over 50% missing values, but no such individuals existed. Seven individuals had given the sample twice, and we used the earliest measurement for those individuals. We removed three individuals with a missing sample date or code, leaving a dataset of 989 individuals. We imputed missing values with a 1/2 minimum value for each metabolite, followed by log transformation using the natural log. After considering missing covariate values, the final dataset consisted of 885 individuals.

### Dimension reduction with KEGG pathways and principal component

We reduced the dimensions of the dataset of 1055 metabolites using an approach consisting of KEGG pathway-based principal component analysis. The overview of the approach is shown in Fig. 1. KEGG pathways^36–38^ were downloaded from the KEGG website^60^ on 2022-10-19. Using identified metabolites, i.e., ones with known chemical identity, their KEGG IDs were mapped to KEGG pathways. Pathways that had at least 10 metabolites are shown in Table S2. All pathways with at least 10 metabolites matching from the Metabolon dataset, plus additionally all metabolites identified as “xenobiotic”, were selected, and for each pathway, a principal component analysis was performed. The cumulative % of variance explained was calculated for the first three principal components. The following principal component was not selected for the subsequent analysis if the difference between two consecutive principal components was $<5\%$. While PCs of each pathway are not correlated, one PC of a pathway can be correlated with PCs from other pathways. Correlations were checked systematically between all PCs, and in case of Pearson correlation $>0.9$, the smaller pathway was removed from the analysis, resulting in a set of 2-3 principal components for each pathway of over 10 metabolites, where all PCs have a correlation of $<0.9$ with PCs in other pathways. The removed pathways are shown in Table S3. The final KEGG-PC dataset consisted of 885 individuals and 91 pathway principal components.

### Hypertension cases and controls definitions

We define hypertension cases as outlined in Table 4, using a combination of ICD-10 codes (for clinical diagnoses), ATC codes (for antihypertensive prescriptions), and self-reported hypertension to exclude prevalent cases from our cohort. To improve diagnostic certainty, we only consider individuals as incident hypertensive if they have at least two recorded hypertension diagnoses or one diagnosis plus a prescription for antihypertensive medication. The date of the first qualifying diagnosis recorded in the electronic health record (EHR) is designated as the date of incident hypertension. First, we divided individuals into hypertension cases, controls and an exclusion set. Hypertension cases were defined as shown in Table 4. Uncertain and the exclusion sets were excluded from cases and controls. Cases were divided into prevalent and incident sets, using the date of the sample and the date of the first diagnosis. Only incident cases (101) and controls (450) were used for further analysis.

**Table 4:** Definition of cases and controls based on electronic health records. Biobank participants were classed as cases, uncertain, exclude and control sets, with exclude and uncertain excluded from both cases and controls. Cases were further divided into prevalent and incident.

Group	Criteria
Cases	$\ge 2$ diagnoses of hypertension (I10, I11, I12 or I13) at least 6 months apart
	Diagnosis of hypertension (I10, I11, I12 or I13) and a prescription of medicine against hypertension (ATC codes C02*, C03*, C04*, C07*, C08* or C09*)
Uncertain	One diagnosis of hypertension (ICD-10 codes I10, I11, I12 or I13) but no other signal of the disease
	Diagnoses of hypertensive disorders in pregnancy, childbirth and the puerperium (ICD-10 codes O10, O11, O13, O14, O15, O16), elevated blood-pressure reading without diagnosis of hypertension (ICD-10 code R03.0), background retinopathy and retinal vascular changes (ICD-10 code H35.0), hypertensive encephalopathy (ICD-10 code I67.4) and secondary hypertension (ICD-10 code I15).
	Have objective measurements of systolic blood pressure over 140 mmHg or diastolic blood pressure over 90 mmHg but no other proof of hypertension
Exclude	No information from electronic health records linking but some answers from the questionnaire
	No information for any diseases
Controls	Not any of the above groups
Incident cases	Cases combined with 1st diagnosis after joining the biobank and uncertain and exclude groups excluded

### Covariate data

For the models, we used the age at the time of the sample as a time scale. For the base model, we considered sex, body mass index (BMI), smoking status, education, residency category and the time of day. For sex, we used the binary biological sex assigned at birth. For the participants who joined and gave the sample until 2017, BMI calculated from measurements at the time of participant recruitment was used. We used a self-reported BMI or BMI from registries for later samples, whichever was closer to the sample date. We used smoking status and education level as the closest self-reported value to the time of the sample. We assigned smoking status “current” to the people reporting themselves as current smokers, “former” to the people reporting as former smokers and “never” for people reporting they have never been smokers. We assigned education class “low” to both people without primary education and with primary education, also with basic education and with vocational education based on basic education. We assigned education class “intermediate” to people with upper secondary education and vocational education based on upper secondary education. We assigned education class “high” to people with Bachelor’s degrees, Master’s degrees and Doctorate degrees or equal diplomas. We assigned the residency category “city” to participants from the five largest cities (Tallinn, Tartu, Narva, Kohtla-Järve, Pärnu). Participants from all other towns were categorised as “town”, and people not living in cities or towns were assigned to the “rural” category based on the population registry. For the time of the day the sample was taken, we used three categories: before 10, 10-15, and after 15. Descriptive statistics of the covariates are given in Table 1.

### Missing values in covariates

Missing values occurred in three variables. There were 24 participants with missing values in the smoking status, 20 participants with missing values in the BMI and one participant with a missing value in the time of day. There was also an overlap among these participants. All the participants with missing values in at least one of these variables were excluded from the analysis. As a result, 27 people were removed, and the questionnaire data of 885 participants remains, including 101 incident cases of hypertension, 334 prevalent cases of hypertension and 450 control cases. In survival models, only incident cases and controls were used.

### Survival models

The dataset used for survival modelling consisted of a metabolomics dataset of 551 individuals, 101 of whom had incident hypertension and 450 had no hypertension diagnosis before joining the biobank and giving the sample and would remain hypertension-free until the end of follow-up period (controls), and 91 principal components of 34 pathways (33 KEGG pathways plus a group of all xenobiotics that also had 3 PCs calculated). The unique metabolites covered in the pathway components were 235. Additional covariate data consisted of age at sampling, time of the day at sampling, hypertension case status, age at diagnosis, death data including age at death, censoring data including age at the end of the follow-up period on 2021-12-31; questionnaire data including sex, BMI, smoking status, education class (low, intermediate, high) and the residency category. No covariates were imputed - people with missing values were removed from the analysis.

CPH models were used to find pathway components associated with the risk of hypertension, where age was used as a time scale. Using age as a time scale corresponds to adjusting for age^40^. Therefore, start refers to the age at the time of giving the sample; stop refers to the age at diagnosis, the age at death or the age at the end of the follow-up period, whichever is the minimum. Participants with prevalent cases of hypertension diagnosed before the time of the sample were excluded from the analysis.

#### Baseline model

First, a CPH was fitted with sex, BMI, smoking status, education class, residency category and time of day included as predictors. The reference levels for categorical variables are “never” for smoking, “male” for sex, “rural area” for residency category, “low” for education class and “before 10” for the time of day. A significance level of 0.05 was used. All possible models that can be constructed by dropping a single model term were compared, and the Chi-Square test was used to find the variable which, when left out, will leave the best model with the lowest Akaike’s information criterion (AIC). AIC is commonly used for model comparisons, and a lower value of the AIC statistic indicates the better model^61^. The assumption of proportionality was checked using scaled Schoenfield residuals.

#### Pathway discovery

For the pathway discovery, a multivariable model was selected in the forward stepwise selection manner. Firstly, we created 91 models, each including the BMI and one of the 91 pathway components as predictors. The pathway component leading to the smallest Benjamini-Hochberg corrected *p*-value in the CPH model was chosen and included in the prediction model. We repeated the process until no additional pathway components were significant at the Benjamini-Hochberg corrected significance level with the FDR controlled at level 0.05.

#### Single metabolite discovery

In order to compare our approach with more traditional approaches, two separate analyses with single metabolites were performed. First, only metabolites that map to KEGG pathways were analysed, in exactly same way as pathway components and with BMI in the baseline model, by creating 212 models each including BMI and one of the 212 metabolites. FDR correction was performed. Secondly, all metabolites with $<20\%$ missing values (1055 metabolites) were also analysed by 1055 separate models including BMI and one of the metabolites. FDR correction was performed.

### Replication in a different cohort

We used another mass-spectrometry dataset (Broad platform, 582 individuals) from the Estonian Biobank for replication^41^. The dataset was not a population-based set of individuals but consisted of people between ages 70 and 79, from whom 187 died 8-14 years after sampling. The individuals were free of hypertensive heart disease (I11.0) according to self-reported data at the time of joining the Estonian Biobank. In 2012, when metabolite profiling was performed, biobank participants had not been linked to electronic health records. As current electronic health linking allows linking diagnoses from 2007, our analysis excluded individuals with hypertension diagnoses (including I11.0) before the sample date. The number of individuals in the full dataset was 582, and from these, 485 had any hypertension diagnosis according to definitions for cases (both incident and prevalent), 22 were uncertain (for example, having just one diagnosis with no further evidence) and 75 are controls. Of the hypertension cases, 162 were disease-free at the time of joining and developed hypertension later (incident cases). Due to some missing values in the covariates and metabolites, the final dataset consisted of 154 incident hypertension cases and 74 controls. The overview of the cohort is shown in Table S6.

### Ethics and data release

The activities of the EstBB are regulated by the Human Genes Research Act, which was adopted in 2000 specifically for the operations of the EstBB. The data collection and research activities of the Estonian Biobank are performed according to the Estonian Human Genes Research Act (HGRA). Informed consent was obtained from the participants upon joining the biobank, allowing their sample and data to be used for further research. Individual-level data analysis in the EstBB was carried out under ethical approval 1.1-12/624 from the Estonian Committee on Bioethics and Human Research (Estonian Ministry of Social Affairs), using data according to release application 3-10/GI/31664 from the Estonian Biobank. All research was performed in accordance with the regulations of the Human Genes Research Act.

## Supplementary Information

Supplementary Information.

## Data Availability

Data used is person-level data of Estonian Biobank participants, which cannot be published by the Estonian law. All data access to the Estonian Biobank’s data must adhere to the informed consent regulations established by the Estonian Committee on Bioethics and Human Research. The datasets analysed in the current study are available from the Estonian Biobank on a reasonable request. To initiate a request for phenotype data, it is necessary to submit a preliminary request to releases@ut.ee. Information about data access, including necessary steps required to access data on the University of Tartu servers can be found at https://genomics.ut.ee/en/content/estonian-biobank Data analysis was performed in R version 3.6.2 (2019-12-12). Code is available on https://doi.org/10.5281/zenodo.14633115
