# Supplementary material for: Pathway level metabolomics analysis identifies carbon metabolism as a key factor of incident hypertension in the Estonian Biobank
Source: Sci Rep. 2025 Mar 12;15:8470. doi: 10.1038/s41598-025-92840-w (PMC11897224; doi:10.1038/s41598-025-92840-w)
Supplement: Supplementary file 1 — Supplementary Information. [file 41598_2025_92840_MOESM1_ESM.pdf]

Pathway level metabolomics analysis identifies carbon metabolism as a key factor of incident hypertension in the Estonian Biobank

Liis Hiie[1,2], Anastassia Kolde[1,2], Natalia Pervjakova[1], Anu Reigo[1], Estonian Biobank Research Team[1], Erik Abner[1], Urmo Võsa[1], Tõnu Esko[1], Krista Fischer[1,2], Priit Palta[1], Jaanika Kronberg[1*]

[1]Estonian Genome Centre, Institute of Genomics, University of Tartu, Riia 23b, Tartu, 51010, Estonia.

[2]Institute of Mathematics and Statistics, University of Tartu, Narva 18, Tartu, 51009, Estonia.

*Corresponding author(s). E-mail(s): jaanika.kronberg@ut.ee; Contributing authors: liis.hiie@ut.ee; anastassia.kolde@ut.ee; natalia.pervjakova@ut.ee; anu.reigo@ut.ee; EstBBresearch@ut.ee; erik.abner@ut.ee; urmo.vosa@ut.ee; tonu.esko@ut.ee; krista.fischer@ut.ee; priit.palta@ut.ee;

1 List of tables

- Supplementary table 1. Mapping metabolites into KEGG pathways
- Supplementary table 2. Cumulative variance explained by principal components of pathways
- Supplementary table 3. Highly correlated principal components
- Supplementary table 4. Results from single pathway component models: BMI and each of the 91 pathway components. Significant single pathway components shown.
- Supplementary table 5. Metabolites in carbon metabolism pathway and their loadings for the 2nd PC
- Supplementary table 6. The descriptive statistics of variables in the replication dataset
- Supplementary table 7. Hazard ratio estimations and p-values for the final Cox proportional hazards model in the replication dataset

- Suppelmentary table 8. Metabolites in carbon metabolism pathway and their loadings for the 3rd PC in the replication dataset
- Supplementary table 9. Results from single metabolite models: BMI and each of the 212 metabolites belonging to pathways. First 12 most relevant metabolite models presented.
- Supplementary table 10. Results from single metabolite models: BMI and each of the 1055 metabolites. First 13 most relevant metabolite models presented.

Supplementary Table S 1 Mapping metabolites into KEGG pathways

Number of metabolites in each pathway	Pathways	Total number of unique KEGG-identified metabolites
≥ 10	33	255
≥ 15	17	199
≥ 20	9	139

Supplementary Table S 2 Cumulative variance explained by principal components of pathways

Pathway	Chem IDs	Cumulative % of variance explained			PCs taken
		PC1	PC2	PC3	
ABC transporters (hsa02010)	43	18.3	27.3	34.7	3
Biosynthesis of aminoacids (hsa01230)	39	23.9	33.7	41.2	3
Biosynthesis of cofactors (hsa01240)	37	13.6	23.5	31.4	3
2Oxocarboxylic acid metabolism (hsa01210)	25	27.3	38.5	46.9	3
Central carbon metabolism cancer (hsa05230)	25	27.5	41.3	50.5	3
Protein digestion absorption (hsa04974)	24	33.8	45.8	52.3	3
Bile secretion (hsa04976)	23	15.3	25.1	34.1	3
DAmino acid metabolism (hsa00470)	21	29.1	42.2	50.8	3
AminoacyltRNA biosynthesis (hsa00970)	20	37.9	51.7	58.2	3
Purine metabolism (hsa00230)	16	17.4	31	42.1	3
Cysteine methionine metabolism (hsa00270)	16	23.3	36.2	46.6	3
Arginine proline metabolism (hsa00330)	16	21.4	31.9	41.8	3
Biosynthesis of unsaturated fatty acids (hsa01040)	16	80.3	86	90.2	2
Mineral absorption (hsa04978)	16	38.9	53.3	60.8	3
Glycine serine threonine metabolism (hsa00260)	15	19.2	31.4	42.2	3
Caffeine metabolism (hsa00232)	14	63	78.3	85.4	3
Carbon metabolism (hsa01200)	14	21.2	36.5	46.7	3
Alanine aspartate glutamate metabolism (hsa00250)	13	21.6	38.5	48.9	3
Histidine metabolism (hsa00340)	13	23.1	37	47.6	3
Phenylalanine metabolism (hsa00360)	13	23.8	40.9	49.9	3
Neuroactive ligandreceptor interaction (hsa04080)	13	24	37.6	47.3	3
Pyrimidine metabolism (hsa00240)	12	17.6	30.9	43.2	3
Taste transduction (hsa04742)	12	26.1	39.7	50.3	3
Arginine biosynthesis (hsa00220)	11	23.7	41.6	51.3	3
Lysine degradation (hsa00310)	11	25.4	37.9	48.2	3
Tryptophan metabolism (hsa00380)	11	30	43	53.7	3
Glyoxylate dicarboxylate metabolism (hsa00630)	11	24.4	43.8	58.3	3
Pantothenate CoA biosynthesis (hsa00770)	11	17.8	31.4	43.7	3
Primary bile acid biosynthesis (hsa00120)	10	32.7	47.2	57.9	3
Steroid hormone biosynthesis (hsa00140)	10	44.7	61.3	74.9	3
Valine leucine isoleucine biosynthesis (hsa00290)	10	40.5	59.3	72	3
Tyrosine metabolism (hsa00350)	10	22.1	35.9	47.5	3
BetaAlanine metabolism (hsa00410)	10	21	35.2	47.6	3
Chemical xenobiotics	24	13.4	25.8	34.4	3

Supplementary Table S 3 Highly correlated principal components.

Larger component	Smaller highly correlated component that is removed from the analysis	Correlation
ABC transporters (hsa02010) PC1	Biosynthesis of aminoacids (hsa01230) PC1	0.92
	Central carbon metabolism cancer (hsa05230) PC1	0.92
	Protein digestion absorption (hsa04974) PC1	0.90
	D-amino acid metabolism (hsa00470) PC1	0.90
Central carbon metabolism cancer (hsa05230) PC2	D-amino acid metabolism (hsa00470) PC2	0.94
Protein digestion absorption (hsa04974) PC2	AminoacyltRNA biosynthesis (hsa00970) PC2	0.99
	Mineral absorption (hsa04978) PC2	0.93
Bile secretion (hsa04976) PC1	Primary bile acid biosynthesis (hsa00120) PC1	0.98
Aminoacyl-tRNA biosynthesis (hsa00970) PC1	Mineral absorption (hsa04978) PC1	0.99
Carbon metabolism (hsa01200) PC1	Alanine aspartate glutamate metabolism (hsa00250) PC1	0.92

Supplementary Table S 4 Results from single pathway component models: BMI and each of the 91 pathway components. Significant single pathway components shown.

Pathway	exp(coef)	adjusted p
Carbon metabolism PC2	1.31	0.0081
Alanine, aspartate, glutamate metabolism PC2	0.81	0.016
Lysine degradation PC2	0.72	0.016
Steroid hormone biosynthesis PC3	1.39	0.016
Central carbon metabolism in cancer PC3	0.81	0.025
Protein digestion and absorption PC2	1.23	0.025
Biosynthesis of amino acids PC2	1.20	0.027
Phenylalanine metabolism PC2	0.82	0.029

Supplementary Table S 5 Metabolites in carbon metabolism pathway and their loadings for the 2nd PC in the discovery dataset

Metabolite	Biochemical subclass	Loading
Glycine	Glycine, Serine and Threonine Metabolism	-0.51
Serine	Glycine, Serine and Threonine Metabolism	-0.51
Citrate	TCA Cycle	-0.32
Alanine	Alanine and Aspartate Metabolism	-0.29
Glutamate	Glutamate Metabolism	0.27
Cysteine	Methionine, Cysteine, SAM and Taurine Metabolism	-0.26
Alpha-ketoglutarate	TCA Cycle	0.24
Gluconate	Food component / plant	-0.15
Aspartate	Alanine and Aspartate Metabolism	0.14
Fumarate	TCA Cycle	-0.14
Glycerate	Glycolysis, Gluconeogenesis and Pyruvate Metabolism	-0.12
Malate	TCA Cycle	-0.10
2-keto-3-deoxy-gluconate	Food component / plant	0.07
Pyruvate	Glycolysis, Gluconeogenesis and Pyruvate Metabolism	-0.07

Supplementary Table S 6 The descriptive statistics of variables in the replication dataset. The statistical significance of differences between incident cases and the control group is assessed using the Welch Two Sample t-test for numeric variables and the Pearson Chi-squared test for categorical variables. A p-value less than 0.05 indicates a statistically significant difference between the two groups.

Variable	Incident cases	Control group	p-value
N	154	74	
Sex, male (%)	22.1	35.1	0.04
Sex, female (%)	77.9	64.9	
Age at sample (mean±sd)	73.5±3	74.2±3	0.05
Average follow-up years	4±4	10±5	$1.01 \cdot 10^{-12}$
BMI (mean±sd)	27.0±4.4	24.7±3.3	$2.14 \cdot 10^{-5}$
Smoking, current (%)	7.8	12.2	
Smoking, former (%)	13.0	14.9	0.49
Smoking, never (%)	79.2	73.0	
Education, low (%)	34.4	32.4	
Education, intermediate (%)	43.5	37.8	0.44
Education, high (%)	22.1	29.7	
Residency category, rural area (%)	23.4	13.5	
Residency category, town (%)	2.6	1.4	0.25
Residency category, city (%)	18.8	17.6	
Residency category, unknown (%)	55.2	67.6	
Time of day, before 10 (%)	16.9	25.7	
Time of day, 10-15 (%)	55.2	58.1	0.09
Time of day, after 15 (%)	27.9	16.2	

Supplementary Table S 7 Hazard ratio estimations and p-values for the final Cox proportional hazards model in the replication dataset

	Model 4	
	exp(coef)	p-value
BMI	1.07	$2.32 \cdot 10^{-4}$
Carbon metabolism (hsa01200) PC3	1.19	$1.06 \cdot 10^{-2}$
AIC	1313.12	

Supplementary Table S 8 Metabolites in carbon metabolism pathway and their loadings for the 3rd PC in the replication dataset

Metabolite	Biochemical subclass	Loading
Aspartate	Alanine and Aspartate Metabolism	-0.62
Glutamate	Glutamate Metabolism	-0.41
Serine	Glycine, Serine and Threonine Metabolism	-0.31
Alanine	Alanine and Aspartate Metabolism	-0.29
Citrate/isocitrate	TCA Cycle	0.28
Fumarate/maleate	TCA Cycle	0.24
Malate	TCA Cycle	0.24
Glycine	Glycine, Serine and Threonine Metabolism	-0.20
Alpha-ketoglutarate	TCA Cycle	0.15
Pyruvate	Glycolysis, Gluconeogenesis and Pyruvate Metabolism	-0.05

Supplementary Table S 9 Results from single metabolite models: BMI and each of the 212 metabolites belonging to pathways. First 12 most relevant metabolite models presented.

Metabolite	Pathway	exp(coef)	p	adjusted p
serine	12 pathways including carbon metabolism	0.19	$5.0 \cdot 10^{-4}$	0.11
retinol (Vitamin A)	Biosynthesis of cofactors	3.87	$1.6 \cdot 10^{-3}$	0.12
N6,N6-dimethyllysine	Lysine degradation	0.48	$1.6 \cdot 10^{-3}$	0.12
glycine	15 pathways including carbon metabolism	0.35	$5.7 \cdot 10^{-3}$	0.20
xanthurenate	Tryptophan metabolism	1.6	$6.4 \cdot 10^{-3}$	0.20
cholesterol	3 pathways including steroid hormone biosynthesis	4.18	$6.4 \cdot 10^{-3}$	0.20
4-hydroxyphenylacetate	2 metabolites including phenylalanine metabolism	0.68	$8.7 \cdot 10^{-3}$	0.20
3-phenylpropionate (hydrocinnamate)	Phenylalanine metabolism	0.80	$9.0 \cdot 10^{-3}$	0.20
phenylacetate	Phenylalanine metabolism	0.74	0.01	0.20
phenylacetylglutamine	Phenylalanine metabolism	0.68	0.01	0.20
alpha-ketoglutaramate*	Alanine aspartate glutamate metabolism	0.29	0.01	0.20
gentisate	Tyrosine metabolism	0.78	0.01	0.20

Supplementary Table S 10 Results from single metabolite models: BMI and each of the 1055 metabolites. First 13 most relevant metabolite models presented.

Metabolite	Pathway	Cexp(coef)	p	adjusted p
X-21733		0.68	$1.4{\cdot}10^{-5}$	0.01
linoleoyl-linoleoyl-glycerol (18:2/18:2) [1]*		1.39	$3.0{\cdot}10^{-4}$	0.10
X-13729		0.66	$3.3{\cdot}10^{-4}$	0.10
3-hydroxybutyroylglycine**		0.74	$4.5{\cdot}10^{-4}$	0.10
serine	12 pathways including carbon metabolism	0.19	$5.0{\cdot}10^{-4}$	0.10
cis-4-decenoylcarnitine (C10:1)		1.84	$7.0{\cdot}10^{-4}$	0.12
S-methylcysteine sulfoxide		0.57	$8.6{\cdot}10^{-4}$	0.12
oleoyl-linoleoyl-glycerol (18:1/18:2) [1]		1.7	$8.8{\cdot}10^{-4}$	0.12
X-12216		0.72	$1.1{\cdot}10^{-3}$	0.12
N6,N6-dimethyllysine	Lysine degradation	0.48	$1.6{\cdot}10^{-3}$	0.14
retinol (Vitamin A)	Biosynthesis of cofactors	3.87	$1.6{\cdot}10^{-3}$	0.14
1-linoleoyl-GPI (18:2)*		2.11	$1.7{\cdot}10^{-3}$	0.14
cinnamoylglycine		0.79	$1.7{\cdot}10^{-3}$	0.14
